# Radical radiation therapy for esophageal carcinoma in situs inversus totalis with the history of surgery for lung cancer: a case report

**DOI:** 10.3389/fonc.2025.1320679

**Published:** 2025-09-03

**Authors:** Yi Chen, Xinmin Xu, Xia Xu, Ming Li, Qunfeng Ma, Shubo Ding

**Affiliations:** ^1^ Department of Radiotherapy, Jinhua Municipal Central Hospital, Jinhua, China; ^2^ Department of Pathology, Jinhua Municipal Central Hospital, Jinhua, China; ^3^ Department of Dental, Jinhua Municipal Central Hospital, Jinhua, China

**Keywords:** situs inversus totalis, radical radiation therapy, esophageal carcinoma, lung carcinoma, multiple primary malignancies

## Abstract

Situs inversus totalis (SIT) is a rare congenital anatomical variation. This case report describes the first instance of a patient with both lung and esophageal cancer along with SIT. Additionally, it presents the first successful radical radiation therapy for esophageal carcinoma with SIT. The patient underwent a left upper lobectomy with lymph node dissection using video-assisted thoracic surgery (VATS) for lung cancer in 2021 and radical radiotherapy for esophageal cancer in 2023.

## 1 Introduction

Esophageal cancer is the sixth leading cause of death and the eighth most common cancer worldwide (1). The five-year survival rate of patients with esophageal cancer is approximately 15 – 25% (2). Situs inversus totalis (SIT) is a rare congenital anatomical variation (3). SIT cases are commonly reported because of the presence of tumors (4). Herein, we present a case report of a patient with SIT who underwent a left upper lobectomy with lymph node dissection using video-assisted thoracic surgery for lung cancer and radical radiotherapy for esophageal cancer.

## 2 Case presentation

### 2.1 Chief complaints

A 58-year-old male patient was admitted to the Department of Cardiothoracic Surgery in May 2023 owing to the choking sensation behind the sternum when swallowing dry for one month.

### 2.2 History of past illness

**Figure 1:**
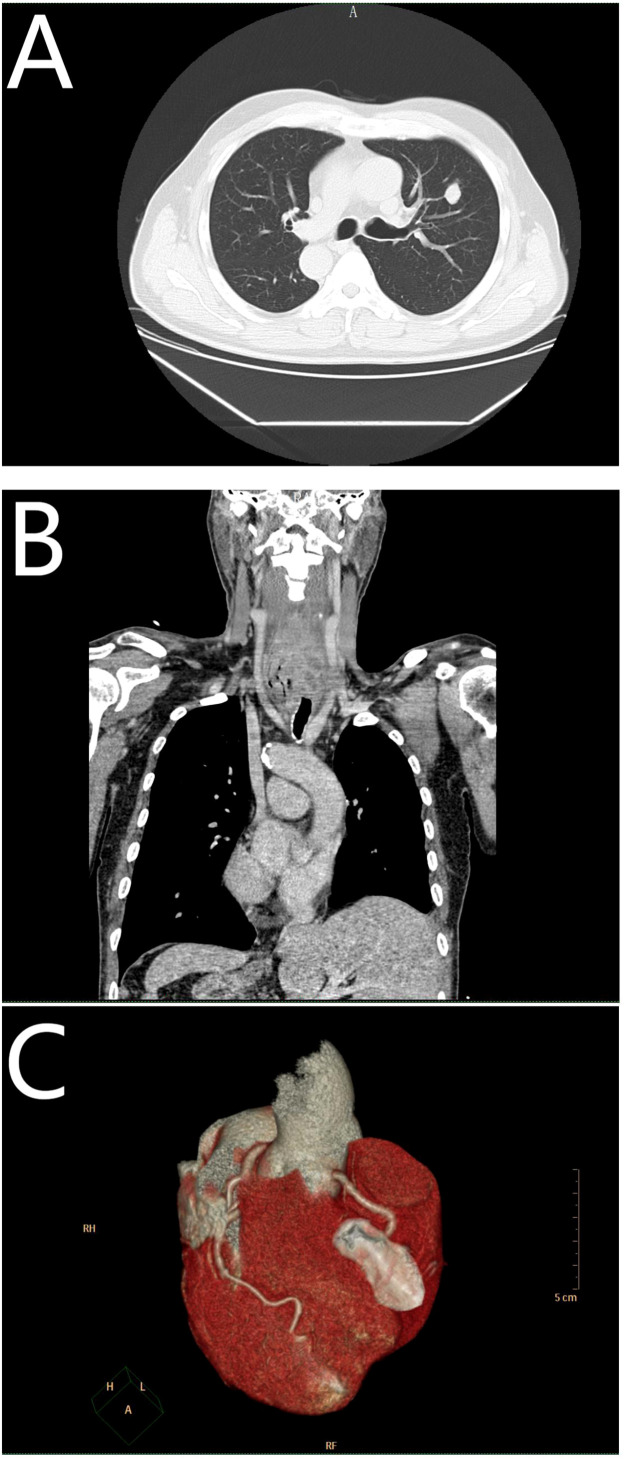
**(A)** Represents a mass of the left lung; **(B)** represents situs inversus totalis (SIT); and **(C)** represents mirror dexiocardia.

**Figure 2:**
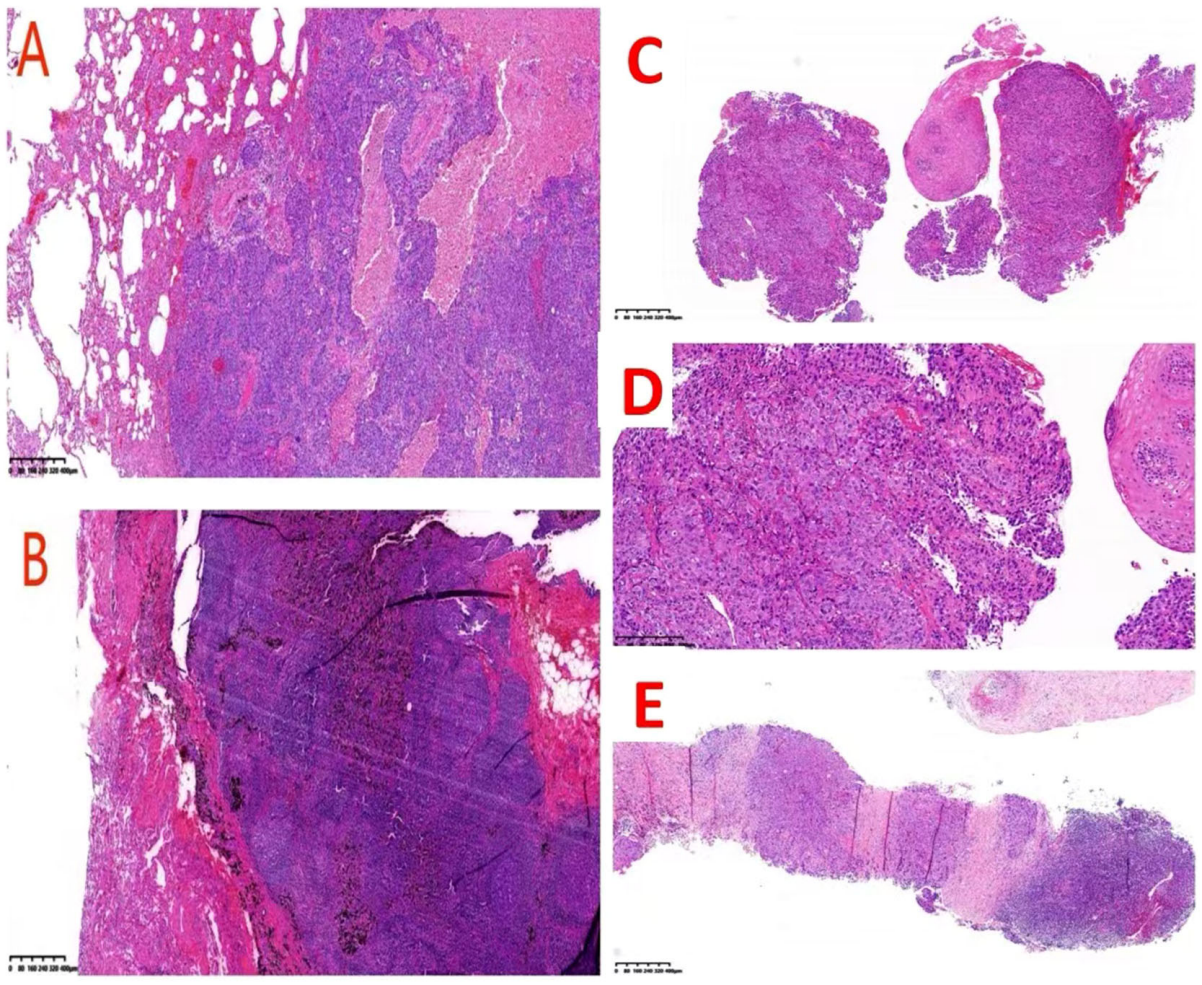
**(A)** Depicts that the pathology of the left upper lung indicates moderately differentiated squamous cell carcinoma(HE, x40); **(B)** depicts that the pathology of the lymph nodes indicates chronic inflammation(HE, x40); **(C)** represents the pathology of the esophagus(HE, x40); **(D)** represents the pathology of the esophagus(HE, x100); node(HE, x40). **(E)** demonstrates the pathology of the left supraclavian lymph node(HE, x40).

In October 2021, the patient underwent a left upper lobectomy with lymph node dissection using VATS. Chest Computed Tomography (CT) showed a mass of the left lung with high density (**Figure 1A**), and a tumor was suspected. All internal organs were inverted (**Figure 1B**), and the presence of mirror dextrocardia was noted (**Figure 1C**). Routine biopsy pathology of left upper lung mass (**Figure 2A**) showed moderately differentiated squamous cell carcinoma (1.7 cm in diameter), with large necrosis, vasculature invasion, nerve invasion (-), and negative bronchial margin. Routine biopsy pathology of two lymph nodes each in groups 2 – 4, 7, 10, 11, and 12 (**Figure 2B**) exhibited chronic inflammation. Immunohistochemistry showed ALK(-), CK5/6(+), CK7(-), Ki-67(+)60%, P40(+), TTF - 1(-), Syn(-), and CD117(-). The patient was diagnosed with squamous cell carcinoma of the lung, which was pathology staged as T1bN0M0, stage IA2 (ninth edition of TNM classification for lung cancer in World Conference on Lung Cancer, 2023). Regular follow-up of the patient showed no signs of recurrent metastasis of lung cancer.

### 2.3 Physical, imaging, and pathological examinations

**Figure 3:**
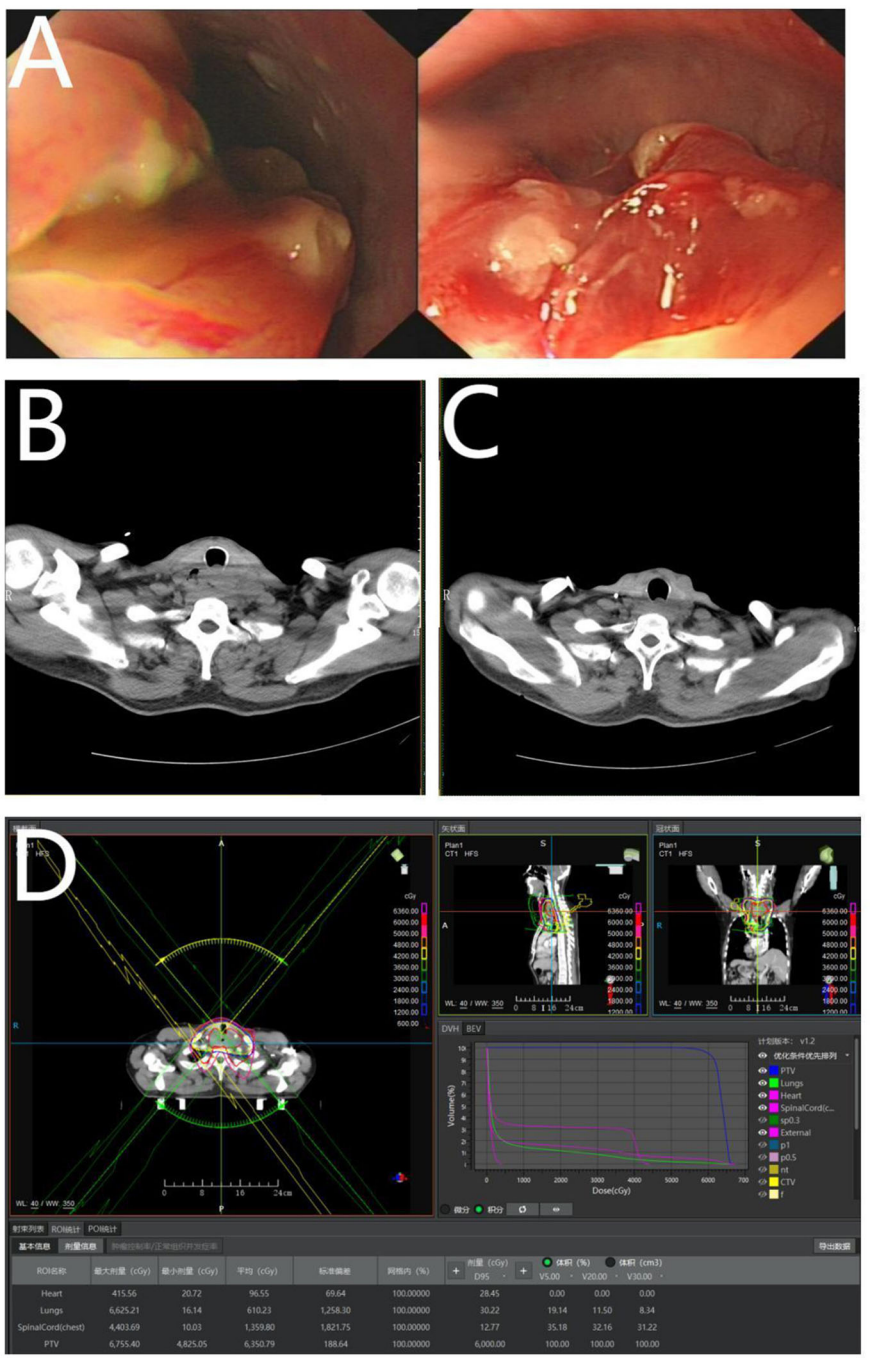
**(A)** Represents a flower-like mass under gastroscopy; **(B)** represents irregular thickening of the upper esophageal wall; **(C)** demonstrates irregular thickening of the upper esophageal wall after radical radiotherapy; and **(D)** represents radical radiotherapy for esophageal cancer.

Physical examination of the patient demonstrated hoarseness in voice and a 2×2 cm enlarged lymph node on the left clavicle. At baseline status, the Karnofsky Performance Status(KPS) score is 90, the Charlson comorbidity index is 4, and the Body mass index is 23.4kg/m². Gastroscopy revealed a flower-like mass localized at 18 cm from the incisors (**Figure 3A**). Esophageal enhanced CT revealed irregular thickening of the upper esophageal wall with luminal stenosis, multiple swollen lymph nodes around it, and invasion of the adjacent trachea. This raised suspicion of a malignant tumor (**Figure 3B**). Routine biopsy pathology demonstrated (esophagus) low differentiated squamous cell carcinoma (**Figures 2C, D**) and (left supraclavian lymph node) metastatic squamous cell carcinoma (**Figure 2E**). Immunohistochemistry results revealed CK7(+), CK5/6(+), P16(-), P40(+), and P53(+).

### 2.4 Final diagnosis

The patient was diagnosed with cervical esophageal squamous cell carcinoma and lymph node metastasis, which was clinically staged as T4bN0M1, stage IVB (Eighth edition of TNM classification for esophageal cancer by the Union for International Cancer Control and American Joint Committee on Cancer, 2017) and was transferred to our department for radiotherapy.

### 2.5 Treatment

The patient underwent radical radiotherapy for esophageal cancer from June 5, 2023, to July 7, 2023. Clinical Target Volume (CTV) included bilateral supraclavicular lymph nodes and para esophageal drainage area 3 cm below the tumor, and Planning Target Volume (PTV) was computed using 0.5 cm of CTV three-dimensional expansion (**Figure 3D**). The prescription dose administered was as follows: 95% PTV = 60 Gray (Gy)/30 F (2 Gy/day, 5 days/week, 6 weeks, total 60 Gy), V5 = 19%, V20 = 12%, V30 = 8%, mean=610 cGy, spinal cord < 4404 cGy, heart < 416 cGy. Five cycles of weekly concurrent chemotherapy of albumin-bound paclitaxel (nab-paclitaxel) (60 mg/m2/day) and nedaplatin (25 mg/m2/day) were performed, during which grade II leukopenia and radiation esophagitis occurred. Considering that the patient’s esophageal cancer stage is IVb, with a KPS score of 90, and his positive attitude toward treatment. After radiotherapy, two additional cycles of chemotherapy of nab-paclitaxel (260 mg/m2/2 days, day 1, 8) and nedaplatin (80 mg/m2/3 days, days 1 - 3) were administered on August 14 and September 5, 2023.

## 3 Follow-up

**Figure 4:**
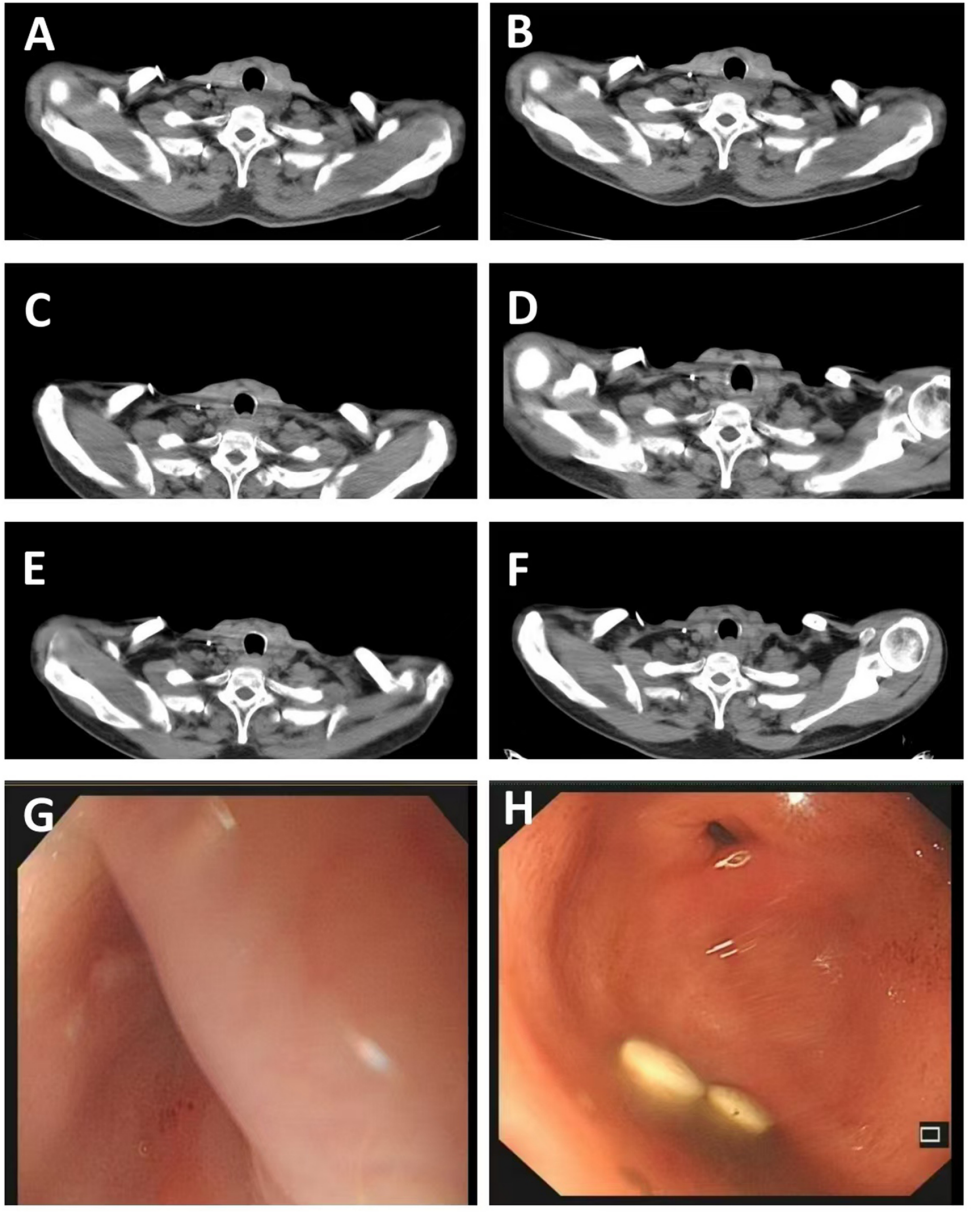
**(A)** Represents irregular thickening of the upper esophageal wall on May 25, 2023; **(B–F)** respectively shows the thickening of the upper esophageal wall during re-examinations on August 12, 2023, October 14, 2023, December 1, 2023, February 22, 2024, and April 9, 2024; **(G, H)** represents smooth mucosa under gastroscopy after radiotherapy on April 9, 2024.

After radical radiotherapy, the patient was followed up for nine months. The 2×2 cm enlarged lymph node on the left clavicle was not observed and there was a significant improvement in choking sensation behind the sternum when swallowing dry. Esophageal-enhanced CT revealed an improvement in the thickening of the wall of the upper esophagus in comparison to the anterior CT (**Figures 3C**, **4A–F**). Gastroscopy showed changes after radiotherapy of the wall of the upper esophagus, but the patient refused to undergo a biopsy (**Figures 4G, H**). There were no signs of tumor recurrence or metastasis.

## 4 Discussion

In this case report, the patient is an esophageal second primary tumor with SIT and prior cancer in the lung. For a patient with stage I or II non-small cell lung cancer (NSCLC), surgical resection remains the treatment of choice on condition that the patient is functionally operable (5). The Chinese expert consensus on cervical lymph node dissection technology of thoracic esophageal cancer (2024 edition), considering the extremely similar ethnic and pathological types of esophageal cancer populations in China and Japan, it is recommended to use JES esophageal cancer lymph node dissection area guidance for surgery and use UICC staging standards for pathological staging. According to the 11th edition of the Japan Esophagus Society staging of esophageal cancer, the patient was clinically staged as T4bN1M0, stage IVA, due to the supraclavicular lymph nodes being classified as the second-station regional lymph nodes in cervical esophageal cancer. However, the patient was clinically staged as T4bN0M1, stage IVB by the Eighth edition of TNM classification for esophageal cancer by the Union for International Cancer Control and American Joint Committee on Cancer, 2017. Based on the above diagnosis, the patient’s good condition, and positive treatment attitude, the patient received radical synchronous radiotherapy and chemotherapy followed by two additional cycles of chemotherapy. The entire treatment process went smoothly, the patient’s discomfort improved, and follow-up showed no recurrence or metastasis of esophageal cancer.

Many survivors of a first primary cancer are at risk of developing a second primary cancer (SPC), with effects on patient prognosis (6). There is a significantly increased risk of secondary primary lung cancer in esophageal cancer survivors(SIR: 2.44, 95% CI: 1.96 - 2.99) (7, 8). The pooled prevalence of lung second primary tumors in patients with esophageal squamous cell carcinoma was 1.8% (95% CI 1.4 - 2.3%). For esophageal second primary tumors in lung cancer patients, the pooled prevalence was 0.2% (95% CI 0.1 - 0.4%) (9). The existence of a prior cancer in esophageal cancer patients was an independent prognostic factor for cancer-specific survival. Esophageal cancer patients with prior prostate cancer and bladder cancer had the best overall survival (OS), while those with prior cancers of the larynx, lung, and bronchus had the worst OS (10). Risk factors for the development of esophageal squamous cell carcinoma include low socioeconomic status, consumption of tobacco, alcohol, hot beverages, and nitrosamines (11). The patient had a long-term history of alcohol consumption before lung cancer surgery, which may also increase the risk of second esophageal cancer.

To date, 21 cases of SIT and lung cancer have been reported in the literature (1).VATS is the first-line strategy as therapy for early-stage, non-small cell lung carcinoma (NSCLC) (12). Kanayama et al. published the first report on video thoracoscopic lobectomy for lung cancer in patients with SIT (13).VATS lobectomy and mediastinal lymph node dissection procedures can be safely performed for NSCLC in patients with SIT by focusing on the symmetrical placement of anatomical structures in the thorax (14).

There have been minimal reports on SIT patients with esophageal cancer (15). Nine cases of SIT with esophageal cancer have been reported in PubMed, and all were surgically treated. Only one study reported neoadjuvant chemoradiotherapy followed by esophagectomy by Aoki et al.in 2011 (16). Our case is the first report of radical radiation therapy in esophageal cancer with SIT. Radiation therapy can be performed for esophageal cancer in patients with SIT by focusing on the right and left organ relocation.

Sun et al. reported a table of information on multiple primary malignancies in patients with situs inversus or situs ambiguous in 2017 (17). Although the occurrence of malignancy in patients with SIT may be coincidental, this report presents a case of both lung and esophagus in a patient with SIT, which may serve as a reference for future research in this area. Radiotherapy can be considered a treatment option for this type of patient.

## Data Availability

The original contributions presented in the study are included in the article/Supplementary Material. Further inquiries can be directed to the corresponding author.
